# High-order harmonics enhancement in laser-induced plasma

**DOI:** 10.1038/s41598-023-41239-6

**Published:** 2023-08-25

**Authors:** Rashid A. Ganeev

**Affiliations:** 1 Laboratory of Nonlinear Optics, Institute of Astronomy, University of Latvia, Riga, 1004 Latvia; 2 Institute of Fundamental and Applied Research, TIIAME National Research University, Kori Niyazov Street 39, 100000 Tashkent, Uzbekistan; 3 Chirchik State Pedagogical University, 104 Amir Temur, 111700 Chirchik, Uzbekistan; 4 Department of Physics, Voronezh State University, Voronezh, 394006 Russia

**Keywords:** Materials science, Optics and photonics, Physics

## Abstract

The methods of enhancement of the strong high-order harmonics of femtosecond pulses in laser-induced plasma are demonstrated. It comprises the application of the four techniques allowing the enhancement of harmonics in different spectral ranges. Among them are the selection of targets for ablation to create the conditions for resonance enhancement of single harmonic, formation of the quasi-phase-matching of a spectrally tunable group of harmonics, application of the two-color pump of plasma, and the formation of nanoparticles-contained plasmas. The number of generated coherent XUV photons increased in the region of single resonantly enhanced harmonic (62 nm) and the shorter-wavelength region (30–50 nm). The above techniques of harmonics enhancement allowed a significant (up to 50 times) growth in a whole harmonic yield in the case of indium plasma. We discuss the reasons preventing the joint implementation of the four methods of harmonics enhancement in the same spectral region.

## Introduction

Various potential applications of short-wavelength radiation in different fields of physics, chemistry, and biology require the development of sources of coherent radiation in the extreme ultraviolet (XUV) range. The high-order harmonics generation (HHG) of ultrashort laser pulses is the most suitable and reliable method allowing the formation of such sources. Among the approaches in this direction are the HHG in gases^1–4^ and HHG during specular reflection of extremely intense laser pulses from the surfaces^5–8^.

The pioneering experiments of HHG in the laser-induced plasmas (LIP) as the media for frequency conversion of laser sources towards XUV, which were carried out in the nineties^9–14^, did not show the perspectives in reliable applications of this method. However, the new trends in this direction demonstrated during the last decade allowed finding a niche in the studies of HHG in plasma using different means, which led to the development of this method of harmonics generation as another approach in formation of the coherent short-wavelength sources. The developments in resonance enhancement of single harmonic, quasi-phase-matching in multi-jet plasmas, applications of quantum dots and large nanoparticles, and other peculiarities distinguished HHG in LIPs from the harmonics generation in gases and during specular reflection from surfaces. The usefulness of this new method of frequency conversion for the spectroscopy of numerous solid elements and the analysis of the ability of harmonics generation during propagation of ultrashort laser pulses through the specially prepared LIPs demonstrated the significance and timeliness of these topics. The experimental and theoretical studies of harmonics generation in plasmas were performed in numerous laboratories worldwide^15–45^.

One can define the most suitable conditions of plasma formation for efficient harmonics generation. This medium should be low-dense (i.e. not exceeding the 5 × 10^17^ cm^−3^ concentration of plasma particles) and weakly ionized (i.e. not exceeding the 8 × 10^16^ cm^−3^ concentration of free electrons). There are a few advantages of this HHG method distinguishing it from other methods of harmonics generation, which allow its application in material science: (a) analysis of the efficiency of this process depending on the structural, morphology, chemical, spectral, etc., properties of the solids; (b) definition of the energy structure of the ionic transitions of ablated solids through the nonlinear optical response of the medium, and investigation of the ionic and atomic transitions possessing strong oscillator strengths through the growth of conversion efficiency of a single harmonic in the vicinity of these transitions; (c) formation of clusters, quantum dots, and large nanoparticles during laser ablation of solids and analysis of their influence on the harmonics generation efficiency with regard to the single atomic/ionic plasmas, as well as ablation of the micro- and nanoparticles already existing on the surfaces of solid materials for HHG studies; (d) formation of the periodically modulated multi-jets plasma structures for the quasi-phase-matching (QPM) of interacting waves in this heterogeneous medium and generation of the groups of enhanced harmonics in different ranges of XUV; (e) application of practically all nonradioactive solid elements of periodic table as the targets for ablation and harmonics generation, contrary to a few gases currently used for HHG, thereby allowing the studies of the spectroscopic characteristics of numerous elements in the XUV range.

Currently, the main concern for coherent XUV sources is a typically rather low generation efficiency (10^–6^–10^–5^) because the HHG still provides a low photon flux. Notice that many applications of such sources require substantially larger brightness in the XUV range. Laser–plasma interaction can be exploited as a promising alternative among the methods of the formation of reliable sources of coherent XUV radiation by achieving the high intensity of generating harmonics, at least in the longer-wavelength part of XUV. The reasons to hope for this are related to the abovementioned advantages of the method of HHG in LIPs.

Though some experiments presented in this paper are available in the literature describing the aspects of harmonics enhancement in LIP, joining various implementations of such enhancement for improving the harmonic flux is an interesting step. No laboratories generate enhanced harmonics using these principles simultaneously due to the phase-matching issues that limit the photon flux of the generated XUV radiation and the experimental complexities associated with the LIP-based HHG. In most cases of HHG in LIPs, the advanced methods of harmonic enhancement were demonstrated separately from each other. Various obstacles like the necessity of maintaining the specific conditions of plasma formation did not allow for the simultaneous application of all methods of harmonics enhancement.

At the same time, there is still a need for improvements in generating strong ultrashort pulses in the XUV range through HHG. One of the approaches is a unification of different methods for harmonics enhancement in LIP, which will allow further growth of the flux of coherent photons in the short-wavelength range. In this paper, the implementation of the two-color pump, quasi-phase-matching, ablated nanoparticles, and resonance-induced growth of harmonic yield is demonstrated both separately and in a single set of experiments. The realization of such an approach increases the areas of XUV pump-probe spectroscopy, applications of coherent XUV photons for the formation of intense attosecond pulses, time-resolved photoemission spectroscopy, X-ray microscopy, and coherent nanoscale imaging.

## Separately demonstrated methods of harmonics enhancement in LIP

**Figure 1:**
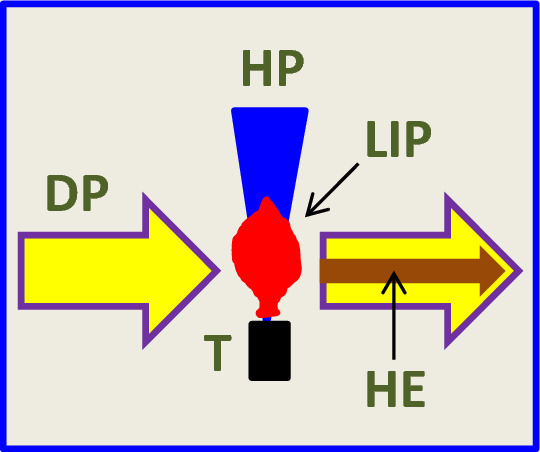
Basic scheme of harmonics generation in laser-induced plasmas. *HP* heating pulse, *T* target, *LIP* laser-induced plasma, *DP* driving pulse, *HE* harmonic emission.

The sketch of HHG in plasma is shown in Fig. 1, which depicts the basic components of the experimental scheme commonly used for the harmonics generation in LIP. The heating pulse (HP) focuses on the target (T) to ablate it and create a LIP. The temporal, energy, and flux characteristics of HP are varied depending on the target to form the suitable plasma for HHG. The femtosecond, picosecond, and nanosecond pulses can be used for the ablation of a target. The fluence of HP can be varied in the range of 0.5–20 J cm^−2^, depending on the properties of ablating material. Then, after some delay (commonly varied in the range of 30–100 ns) from the beginning of ablation, the driving pulse (DP) focuses inside the LIP from the orthogonal direction to generate a harmonic emission (HE). The optimal delay between HP and DP is determined by the distance between the DP propagation and target, the velocity of LIP, and various characteristics of HPs. The wavelengths of DP are 800 and 1310 (or in some cases 1420) nm. The intensity of DP is varied in the range between 5 × 10^13^ and 5 × 10^14^ W cm^2^, while the pulse duration can be varied in a broad range (4–300 fs). The harmonic emission is registered by an XUV spectrometer^46^.

As mentioned, previous studies demonstrated four methods of harmonic enhancement in LIPs: two-color-pump-induced enhancement, quasi-phase-matching-induced enhancement, resonance-induced enhancement, and nanoparticles-induced enhancement. In this section, four processes are demonstrated separately from each other. The physical mechanisms of such types of enhancement are not explained in detail in this paper since those processes were analyzed elsewhere^47–50^.

### Two-color-pump-induced enhancement of harmonics

**Figure 2:**
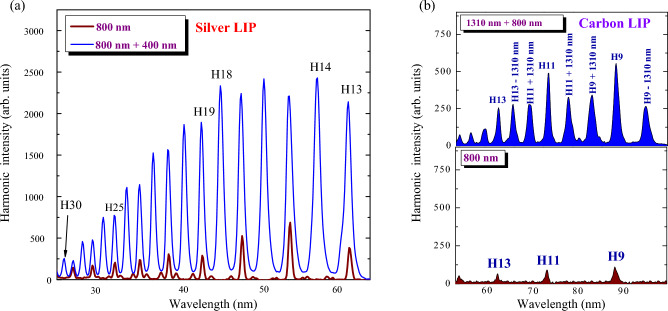
Two-color-pump-induced enhancement of harmonics. (**a**) Application of single-color pump (800 nm, brown curve) and two-color pump (800 nm + 400 nm, blue curve) for harmonics generation in silver plasma. (**b**) Generation of odd harmonics during 800 nm pump (bottom panel) and sum and difference frequencies alongside with odd harmonics of 800 nm pump during pumping of carbon plasma by incommensurate waves (800 nm + 1310 nm, upper panel).

The typical harmonic spectra during single-color (800 nm) and two-color (800 nm + 400 nm) pumps of silver LIP are shown in Fig. 2a. The intensity of the higher orders of harmonics in the plateau region gradually decreases during the single-color pump (brown curve). In Fig. 2a and the following figures, the Nth harmonic order is depicted as HN. Particularly, H13 denotes the 13th harmonic order of photon energy 13 × E, where E ≈ 1.5 eV for the DP at a wavelength ~ 800 nm.

This harmonic yield is notably enhanced by using the two-color pump of the same LIP (Fig. 2a, blue curve). The odd and even harmonics in that case were three to four times stronger than the odd harmonics generated during the single-color pump. These two curves demonstrate the enhancement of harmonics in the case of the pump of LIP by commensurate waves, i.e. the fundamental radiation (800 nm) and its second harmonic (400 nm) produced in the barium borate (BBO) placed on the path of 800 nm beam inside the vacuum chamber.

Figure 2b, similar to Fig. 2a, demonstrates the advantages of using the two-color pump of LIP in the case of incommensurate waves, i.e. the waves, which cannot be considered as the integers to each other. The bottom panel shows the odd harmonics generation using the pump of carbon plasma by 800 nm pulses. The addition of the 1310 nm pump to the 800 nm pump allows for demonstrating the enhancement of odd harmonics, as well as the generation of sum and difference frequencies of comparable intensities.

The mechanisms of two-color pump-induced enhancement of harmonics were previously described during a demonstration of HHG in gases, which suggested that strong harmonic generation, in that case, is possible due to the (i) higher ionization rate compared with a single-color pump when only one wave interacts with the matter formation of a quasi-linear field and (ii) selection of a short quantum path component, which has a denser electron wave packet^51^.

### Quasi-phase-matching induced enhancement of a group of harmonics

Another phenomenon leading to the enhancement of a group of harmonics is the effect of quasi-phase-matching between the interacting waves. QPM in the XUV range has first been demonstrated in the case of multi-jet gases^52^. This is an attractive approach for resolving the phase-mismatch problem during HHG in the XUV range.

Meanwhile, QPM in LIP may offer a few prospective applications, which hardly can be realized using gas medium: (a) easy manipulation of the characteristics of multi-jet plasmas using the multi-slit masks (MSM) (e.g., the width of a single jet, the distance between jets, electron concentration, etc.); (b) definition of the electron concentration in LIPs through the measured values of maximally enhanced harmonics and the coherence lengths corresponding to the distance between the jets at which the group of enhanced harmonics was observed; (c) implementation of QPM using the perforated ablation beams, perforated targets, or interfering HPs; (d) easy tuning of maximally enhanced groups of harmonics by either changing the MSMs with different slit sizes or changing the fluence of HP on the target surface; (e) amendments in the fluencies of converted XUV radiation. Presently, none of these prospective applications were reported using the multi-jet gas media.

The pumping scheme using the MSM was as follows. The cylindrical lens focused the picosecond heating beam on the target surface thus allowing the formation of homogeneous extended plasma. Once the MSM became introduced on the path of the heating beam, the homogeneous expended plasma divided thus leading to the formation of the multi-jet plasma. The femtosecond driving beam then propagated through this multi-jet plasma. The confocal parameter of the femtosecond driving beam (7 mm) was larger than the whole length of the plasma (5 mm). The 6-jet plasma, 4-jet plasma, and single-jet plasma were used in these experiments. The change in the number of jets was performed by shielding the slits. The experiments with a variable number of slits were performed to demonstrate the quadratic dependence of the harmonic yield on the number of jets. The variable MSMs, which distinguish from each other by the sizes of slits, were used to demonstrate the QPM effect in different spectral ranges.

The slits were cut on the 0.2 mm thick stainless steel plates using the micromachining technique. The size of the open part was equal to the size of the closed parts. The slit sizes of 0.2, 0.3, 0.35, 0.5, and 0.8 mm were applied for these studies to determine the best conditions of QPM in different spectral ranges and at different fluencies of the heating picosecond pulses. The distance between plasma jets was equal to the slit sizes. No detectable diffraction of the heating beam on the target surface was observed since the distance between the MSM and the target was relatively small (~ 200 mm).

**Figure 3:**
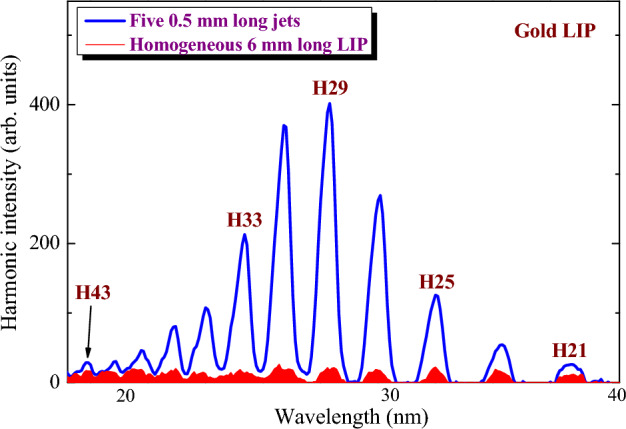
Harmonic spectra generated in the 6 mm long gold plasma (filled red curve) and five 0.5 mm long plasma jets (blue curve). Gold plasma was pumped by 800 nm DP.

Figure 3 shows the distinction in the harmonic generation in the cases of homogeneous extended plasma and heterogeneous (multi-jet) LIP. In this figure, the harmonic spectra from the 6 mm long homogeneous gold plasma (filled red curve) and five 0.5 mm long gold plasma jets (blue line) are presented. One can see a significant enhancement of a group of harmonics centered at H29 (27.6 nm) of the 800 nm pump.

The mechanism of enhancement of harmonics in the multi-jet medium (gases, plasmas) is as follows. The phase mismatch between interacting pulses diminishes the harmonic yield. This process is related to the appearance of the difference in the wave numbers (Δ*k*) of the harmonic field and the laser-induced polarization. The main mechanism here is the dispersion of the medium, which enhances by the presence of free electrons. At some distance from the beginning of the medium called coherence length, the phase shift between interacting waves of fundamental and harmonic emissions becomes close to *π*. At a distance longer than the coherence length (*L*_coh_ = *π/*Δ*k*) the destructive conversion of harmonic photons back to the fundamental emission starts to play a decisive role at the conditions when the newly generated harmonics photons being in reverse phase compensate for the earlier generated photons. This decrease in harmonic yield can be notably diminished by so-called methods of quasi-phase-matching, which allow overpass of the reversion of harmonic conversion efficiency. One such method is the use of multiple gas jets^52–54^ and plasmas jets^48,55^. The separation of the extended medium by a set of separated gas or plasma jets of the sizes of *L*_coh_ resolves the problem of phase-mismatching.

### Resonance-induced enhancement of single harmonic

During HHG from a few LIPs, the intensity of some harmonic order becomes abnormally larger than the one of neighboring harmonics^56^. This phenomenon is known as resonantly enhanced single harmonic generation, and the corresponding harmonic can be dubbed as resonant harmonic (RH).

**Figure 4:**
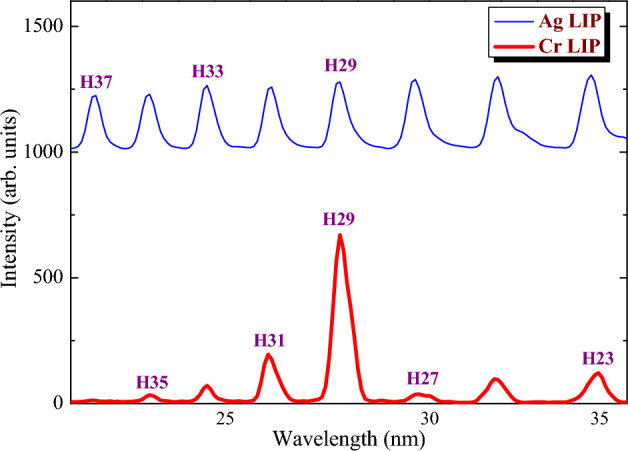
Comparative spectra of the harmonics generated in Ag (upper curve) and Cr (bottom curve) LIPs using the 800 nm pump. The curve for Ag LIP is shifted along the *Y*-axis for better visibility of the featureless plateau-like distribution of harmonics and comparison with the strongly modulated harmonic spectrum from Cr LIP.

The studies presented in Fig. 4 allow demonstrating a difference in harmonics distribution in the case of Cr and Ag plasmas due to the realization of a resonance enhancement in the former LIP. Earlier, HHG in Cr LIP using a single-color 800 nm pump was compared with the application of a two-color pump (1310 nm + 650 nm), and strongly enhanced harmonic was observed in both cases in the region of 27 nm^57^. In the present case (800 nm DP), a significant suppression of the 27th harmonic is followed by the enhancement of the 29th harmonic in the abovementioned spectral region (Fig. 4, bottom curve). One can see that only H29 (and partially H31) was enhanced, while in the case of silver plasma, no enhancement of harmonics was demonstrated (Fig. 4, upper curve). In the latter case, the featureless plateau-like distribution of harmonics was observed up to the cutoff energy (H59, not shown in this figure). These comparative studies show that there is some transition corresponding to the group of resonances of the Cr II spectrum in the region of 27 nm, which strongly affects the nonlinear optical response of this plasma. This is a clear example of the nonlinear spectroscopy of plasma using HHG.

Several mechanisms of resonant enhancement have been discussed in the literature, generally all involving an intermediate, resonant, step in the semiclassical model. The generation of high harmonics with frequencies close to that of the transition from the ground state to an autoionizing state (AIS) of the generating particle was experimentally investigated in plasma media^58^ and in noble gases^59^. Several theories describing HHG enhancement based on the specific properties of AIS were developed^18–20,60–63^. Particularly, in Ref.^19^ a four-step resonant HHG model was suggested. The overview of different resonance-related processes during HHG in LIPs is presented in Ref.^64^.

### Nanoparticles-induced enhancement of harmonics

**Figure 5:**
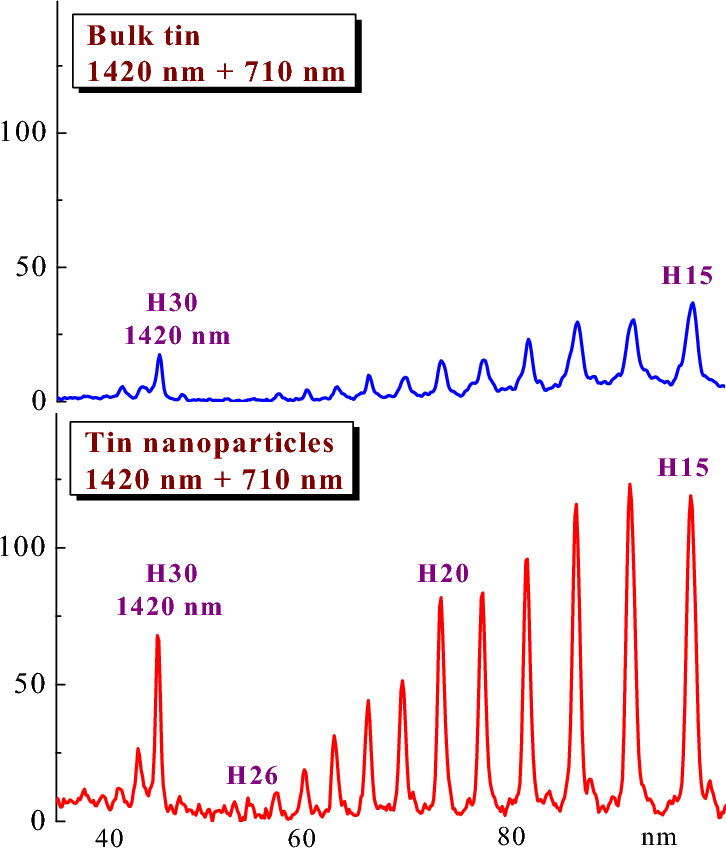
Harmonic spectra in the cases of the two-color pump of bulk tin plasma (upper panel, 1420 nm + 710 nm) and tin nanoparticle plasma (bottom panel, 1420 nm + 710 nm).

The application of nanoparticles instead of single particles (atoms and ions) can enhance the harmonic yield from the same elemental state of plasma. Figure 5 demonstrates such an enhancement of harmonic emission from the 15 nm nanoparticles-contained LIP compared with the plasma containing atomic species of the same elemental state.

Here are shown the harmonic spectra in the cases of the two-color pump of bulk tin plasma (upper panel, 1420 nm + 710 nm pump) and tin nanoparticle plasma (bottom panel, 1420 nm + 710 nm pump). A fourfold enhancement of harmonics in the latter case was demonstrated. A similar conclusion about the growth of harmonic yield from Sn nanoparticles was reported in Ref.^65^ where the tuning of DP allowed determining the best conditions for the resonance enhancement of a single harmonic in the vicinity of 47 nm and the growth of yield of a whole group of harmonics in the case of HHG in Sn nanoparticles.

The nonlinear optical features of nanoparticles attracted great attention particularly due to their applications in HHG^66^. A relatively strong enhancement of HHG yield (up to 20×) was achieved in the LIPs containing spherical nanoparticles of gold targets ablated in a vacuum using the picosecond HP^67^. It was shown that efficient generation of harmonics can also be achieved in the plasmas containing quantum dots of semiconductor materials^68,69^.

## Combining different mechanisms of harmonics enhancement in LIP

Below, the merger of the four above-analyzed methods of harmonics enhancement in LIP is demonstrated in a single set of experiments satisfying the requirements for the enhancement of harmonic yield in the XUV range using (a) quasi-phase-matching, (b) two-color pump, (c) resonance-induced processes, and (d) nanoparticles medium. The simultaneous application of (a–d) methods allows for combining the achievements of each separate method thus further increasing the harmonic yield and broadening the ability in investigation of materials. Notice that the enhancement factor in the case of the merger of those methods does not correspond to the sum of separated methods of enhancement since the optimal conditions of plasma formation in the latter cases did not coincide with the same in the case of the merger of all methods of harmonics enhancement. Below we demonstrate the enhancement of a single harmonic using the RH effect, two-color pump, and nanoparticle-induced enhancement, while the fourth method (QPM) was demonstrated in another spectral range. In the discussion section, we analyze the problems arising during shifting the QPM-induced enhancement toward the position of RH.

**Figure 6:**
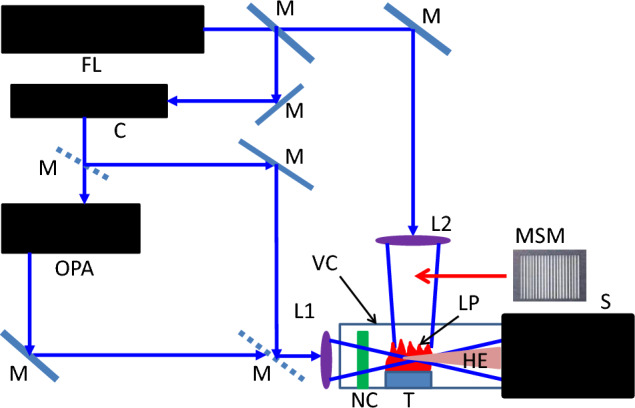
Scheme of experiments. *FL* femtosecond laser, *C* compressor of pulses, *OPA* optical parametric amplifier, *M* mirrors, *VC* vacuum chamber, *S* XUV spectrometer, *MSM* multi-slit mask, *L1* spherical lens; *L2* cylindrical lens, *NC* barium borate (BBO) nonlinear crystal, *T* target, *HE* harmonic emission.

Figure 6 presents the scheme for harmonics generation when all the above-mentioned mechanisms of the enhancement of coherent XUV emission can be implemented in a single set of measurements. It allows the two-color pump of LIP, modification of extended plasma towards the formation of multi-jet structure using MSM, application of the metal (indium) allowing the generation of extremely strong resonance-enhanced harmonic, and application of the nanoparticles of this metal for further enhancement of harmonic yield.

The bulk indium plate was placed in a vacuum chamber, which allowed the realization of the single harmonic enhancement. To demonstrate the nanoparticles-induced enhancement, the bulk In target was replaced by a target comprising 20 nm nanoparticles of indium. The vacuum in the chamber was 10^–5^ mbar. Ti: sapphire laser generating at the wavelength 800 nm was used as a driving laser. In these experiments, the 150 ps uncompressed laser pulses and 64 fs compressed laser pulses at a 10 Hz pulse repetition rate were used. The 150 ps pulses were split by a beam splitter into a first beam to provide the required pulse energy of the picosecond laser pulses for laser ablation and a second beam for harmonics generation. The first beam was focused by a cylindrical lens onto an indium solid target or indium nanoparticles-containing target mounted in a vacuum chamber to provide the fluence of 1 J cm^−2^ on the target surface for generating the 5 mm long LIP. The insertion of MSM on the pass of this uncompressed beam allowed a division of the extended plasma and formation of the multi-jet LIP required for the realization of the conditions of QPM between the DP and the group of harmonics. Depending on the length of a single plasma jet, the maximally enhanced harmonic in this group of harmonics was tuned along the XUV range.

The second laser beam was compressed in a grating compressor to provide the 64 fs pulses. This radiation was directed to the formed LIP in the vacuum chamber containing the target or used for the pump of the optical parametric amplifier. In the first case, the 64 fs pulses were focused inside the LIP using the spherical focusing lens. The two-color pump of plasma was implemented by inserting a thin (0.3 mm) BBO crystal inside the vacuum chamber on the pass of the focused driving beam to generate s second harmonic radiation (400 nm, Fig. 6) at a conversion efficiency of 4%. The resulting 400 nm driving laser pulses interacted with the plasma particles, alongside the 800 nm pulses to generate the odd and even harmonics. In the second case, the near-infrared signal pulses (*λ* = 1310 nm) from the optical parametric amplifier were used as the DPs for harmonics generation. In that case, the two-color pump was also used to allow the generation of the tunable odd and even harmonics to determine the conditions for the maximal enhancement factor of the RH and other harmonics. All below-described experiments were carried out using the 1310 nm DPs.

To generate RH, the target was selected to achieve the multiphoton resonance of the driving radiation with the target’s ionic transition for a relatively low (13th) order of 800 nm radiation (wavelength 61.5 nm). The 19.92-eV ionic transition of indium 4*d*^10^5*s*^2 1^*S*_0_ → 4*d*^9^5*s*^2^5*p*(^2^*D*) ^1^*P*_1_ from the ground state to AIS has a wavelength (*λ* = 62.2 nm), which is close to that of the 13th harmonic of 800 nm radiation. The oscillator strength of this ionic transition substantially exceeds those of the neighboring transitions in this spectral region^70^.

The generated harmonics and driving beam propagated through the input slit of the XUV spectrometer and reflected from the cylindrical gold-coated mirror at a 4° angle of interaction. Then these emissions were spectrally dispersed by a flat-field grating, and the short-wavelength radiation was detected by a microchannel plate. The harmonic distribution appearing on a phosphor screen of the microchannel plate was recorded by a CCD camera.

**Figure 7:**
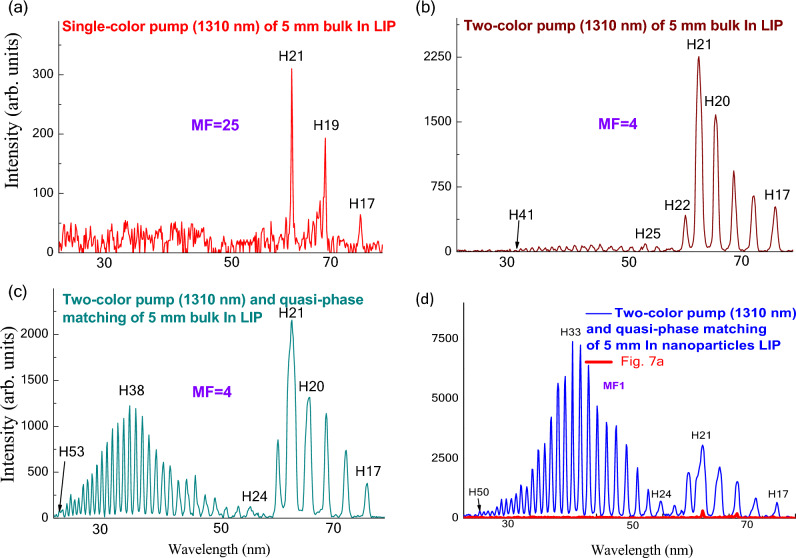
Harmonic spectra in the case of (**a**) single-color pump (1310 nm) of the plasma produced on the 5 mm long bulk indium target, (**b**) two-color pump (1310 nm + 655 nm) of the plasma produced on the 5 mm long bulk indium target, (**c**) two-color pump (1310 nm + 655 nm) and quasi-phase-matching conditions in the perforated plasma produced on the 5 mm long bulk indium target, and (**d**) two-color pump (1310 nm + 655 nm) and quasi-phase-matching conditions in the perforated plasma produced on the 5 mm long indium nanoparticles-contained target. MF corresponds to the magnification factor of curves with regard to the curve (**d**). The red curve in (**d**) repeats the one shown in (**a**) for a visual comparison of the harmonics enhancement after the implementation of all methods.

The step-by-step procedure of the implementation of the four above-described methods of harmonics enhancement is shown in Fig. 7. The application of a single-color pump (1310 nm) of the extended (5 mm) plasma produced on the surface of bulk indium target allowed a generation of weak odd harmonics (H17–H21) showing the unusual distribution (Fig. 7a). One can see the enhancement of RH (H21) in the wavelength range close to the strong ionic transition (62.2 nm). This harmonic (*λ* = 62.4 nm) almost coincided with the abovementioned ionic resonance of indium, which led to a stronger intensity of H21 compared with the lower-order harmonics. There were no harmonics above this order in the case of the 5 mm long plasma.

The significantly stronger harmonic spectrum, and particularly H21, appeared in the case of the two-color pump (1310 nm + 655 nm) of the 5 mm long plasma produced on the bulk indium (Fig. 7b) once the 0.5 mm long BBO was inserted on the pass of the 1310 nm radiation allowing the generation of 655 nm radiation at the 11% conversion efficiency. Thus the application of two methods (resonance enhancement and two-color pump) allowed a notable (approximately sixfold) growth of the RH harmonic yield at similar conditions of laser-matter interaction despite the small ratio of the intensity of the second field (655 nm) with regard to the fundamental radiation (1:8). One can see the appearance of weak odd and even harmonics in the shorter wavelength range (i.e. for the harmonic orders exceeding H22). The harmonic cutoff was in the range of H41 (*λ* ≈ 32 nm).

The addition of the fourth method (QPM) resulted in notably stronger harmonic emission in a broad shorter-wavelength spectral range (Fig. 7c). Though the intensity of the lower-order harmonics remained approximately the same as in the previous case (compare the H17–H22 in Fig. 7b,c), a significant growth (up to 15 ×) of the higher-order harmonics (H24–H53) was obtained. The formation of perforated plasma (i.e. multi-jet LIP) by introducing MSM on the pass of HP allowed for the creation of the conditions when the phase matching for a large group of harmonics centered at H38 of 1310 nm pump (*λ* ≈ 34.5 nm) led to the heterogeneous growth of the yield of those harmonics.

The relation between the maximally QPM-enhanced harmonic order (*q*_QPM_), sizes of single plasma jet (*l*), and electron density in LIP (*N*_e_) is *q*_QPM_ ∝ (*l* × *N*_e_)^−1^^48,71^. This simple relation allows calculating the maximally enhanced harmonics by estimating the electron concentration and knowing the thickness of a single jet in the multi-jet LIP. The former parameter approximately corresponds to the width of a single slit, which is commonly varied between 0.2 and 0.8 mm. For the plasma jets of different sizes, the maximally enhanced harmonics will be tuned along the XUV spectrum. Similarly, a decrease in the fluence of HP causes a decrease in electron density, which moves *q*_QPM_ towards the shorter-wavelength region of XUV. The intensity of harmonics in the longer-wavelength range of generation remains almost unchanged during variation of *l* and *N*_e_, as was confirmed once comparing Fig. 7b,c. In the meantime, the intensities of RH (H21) and *q*_QPM_ (H38) became comparable to each other (H38:H21 ≈ 1:2). Thus this method of enhancement was not able to enhance the lower-order harmonics. The reasons for that will be discussed in the discussion section. Meanwhile, the comparison of the same harmonics at the conditions of QPM using MSM with the 0.4 mm sizes of the single slit (Fig. 7c:1:2) and of extended In LIP (Fig. 7b:1:30) shows the effectiveness of this method of harmonics enhancement in the shorter-wavelength region. Similar amendment of harmonic yield in the shorter wavelength range in the case of multi-jet In plasma^72^ have demonstrated the tuning of *q*_QPM_ depending on the conditions of target ablation.

The replacement of the bulk target by the one comprised of the nanoparticles of the same elemental consistency at the same conditions of the experiment as those described in Fig. 7c allowed for observation of the modification of enhancement factor attributed to the presence of nanoparticles in LIP. In that case, a rather small enhancement (~ 1.2) of harmonic yield was observed for RH and nearby longer-wavelength harmonics (Fig. 7d) compared with the case of ablation of the bulk target (Fig. 7c) due to the necessity of the simultaneous maintenance of the optimal conditions for other processes (resonance enhancement, two-color pump, and QPM). Meanwhile, the notable growth of harmonic yield (~ 7×) was obtained in the shorter-wavelength region, particularly for the group of QPM-enhanced harmonics generated in the indium nanoparticle plasma (compare the intensities of the shorter-wavelength harmonics in Fig. 7c,d). Thus the use of nanoparticle-containing targets results in a decrease of material-specific properties like resonance enhancement of single harmonic on the enhancement factor. At the same time, past and present studies show that in contrast to single harmonic resonance, the intensity of all harmonic orders is increased in the case of nanoparticle-containing plasmas.

The application of nanoparticles in most cases leads to a multi-fold growth of harmonic emission (up to a few tens^31,45,49^). However, this is true only in the case when optimization of plasma conditions is carried out without the consideration of other processes causing the enhancement of harmonic yield. Because of this in present studies, the enhancement factor related to the application of multi-particle species for HHG was relatively small (1.2×) for the harmonics in the vicinity of RH and large (~ 7×) for the harmonics related to the QPM-induced enhancement.

As it was mentioned, the tuning of *q*_QPM_ depends on the conditions of target ablation. The same can be attributed to the properties of the target to form the suitable plasma during laser ablation. In the case of the ablation of indium nanoparticles, one has to use a larger fluence of HP, which causes the growth of concentration of the free electrons. The abovementioned relation for QPM predicts in that case a shift of maximally enhanced harmonic towards to longer wavelength region, which was observed in our experiments when *q*_QPM_ was shifted from H38 to H33 (compare Fig. 7c,d).

The reported experimental and theoretical studies, which apply to HHG from gas clusters^73–77^, do not explain well the HHG from nanoparticles. The method to calculate HHG from nanoparticles was developed in Ref.^31^. With the help of semi-classical arguments, the harmonic enhancement in the case of nanoparticles-contained plasma has been analyzed. It was shown that enhancement for a particular harmonic order is almost independent of the nanoparticle size. In the case of gas clusters, the mechanism of harmonics enhancement was attributed to the involvement of the local field due to confinement effects. However, it is probably not correct in the case of large multi-particle species like nanoparticles when inner atoms do not participate in harmonics generation. Meanwhile, the large sizes of nanoparticles allow for increasing the cross-section of the recombination of the accelerated electron with the parent particle during the three-step process of harmonics generation. Further studies are required for understanding the mechanisms of harmonics enhancement in the case of nanoparticles.

Parameter MF shown in Fig. 7 refers to the approximate magnification factors of the curves (a–c) with regard to the curve shown in Fig. 7d. Apart from the large increase of MF (25×) from the point of view of comparison of the maximal intensities of generating harmonics (compare Fig. 7a,d) once can admit a significant growth of the short-wavelength generating components in the studied region of XUV. The red curve in Fig. 7d repeats the one shown in Fig. 7a for a visual comparison of the harmonics enhancement after the implementation of all methods. The enhancement factor for the RH in that case was ~ 8. Meanwhile, no high-order harmonics above H21 were observed in the case shown in Fig. 7a. Those harmonics appeared only in the case of the two-color pump (Fig. 7b). So the comparison of the QPM-induced enhancement can be carried out by comparing the harmonic spectra shown in Fig. 7b,d. Particularly, the maximal QPM-enhanced harmonic (*q*_QPM_ = H33, Fig. 7d) was more than 100 times stronger than the same harmonic shown in Fig. 7b.

The harmonic distribution presented in Fig. 7d demonstrates a joint implementation of four methods of harmonics enhancement: resonance-induced enhancement of single harmonic (versus homogeneous decay of harmonics in most plasma plumes), two-color pump (versus single-color pump) of plasma, the pump of nanoparticles instead of atoms and ions, quasi-phase-matching of a group of harmonics in the shorter-wavelength range (versus featureless decay of the weak higher-order harmonic). The two latter processes allowed for achieving a significant enhancement of a large group of harmonics, which is comparable with the resonance mechanism of single harmonic enhancement. Meanwhile, QPM was not so effective in the region of RH, which will be discussed later.

HHG in LIP differs from the same process occurring in gases by the use of numerous elements of the periodic table being presented in the form of an ablation plume, as well as by the option of simultaneous realization of different methods of harmonics enhancement. The novelty of the present approach is a demonstration of the combination of a few methods of harmonics enhancement in LIPs in different ranges of XUV. Such an approach results in the enhancement of the overall photon flux in XUV range and the high conversion efficiency of the resonantly enhanced harmonic (~ 10^–4^^56^). Such methods can be applied in the case of a few other metal targets allowing the generation of RH (Mn, Sn, As, Se, Te, Cr, Mo).

## Discussion

The joint implementation of only two mechanisms of harmonics enhancement is not a new effect, since examples of such applications were frequently reported in both gas and plasma HHG studies. It is hard to achieve such a merger of all four methods of enhancement in various laser plasma species. The present study aimed to demonstrate the advanced features of LIP as the medium allowing modifying its properties for the enhancement of the HHG, which cannot be realized in gas jets.

Gas is a medium, which has a limited number of options to modify the harmonic generation process compared with the LIP, which may contain the neutrals, singly- and doubly-charged ions, free electrons, and multi-particle species (large molecules, clusters, quantum dots, nanoparticles, and even microparticles). Notice that it is exceptionally difficult to demonstrate the RH effect in gases. Consequently, the quasi-phase-matching in a set of a few gas jets cannot be combined with the resonance enhancement of a single harmonic. Similarly, the harmonics enhancement through the application of the clusters of gaseous atoms cannot be compared with the plasmas containing carefully separated large molecules, clusters, quantum dots, and large nanoparticles. The same is true for the two-color pump of gases, which is frequently used during gas HHG experiments but cannot be merged with other above-mentioned methods of enhancement. Thus, the LIP has larger perspectives in the demonstration of enhancement techniques compared to ordinary gases.

The demonstrated method of the merger of some enhancement processes allowed for the increase of the number of harmonic photons in the same spectral region (62 nm). Notice again that the one of methods (QPM) was not able to enhance the harmonics in this longer-wavelength region of XUV. Simultaneously, this research is also about material science, which demonstrates that the notably larger options in the manipulation of plasma characteristics allow for determining some specific (i.e., spectral, morphological, spatial, etc.) features of practically any solid material, which can be transformed toward the plasma state. The suggested method is just an example of how different approaches in plasma manipulations can lead to the growth of the yield of coherent XUV emission in different spectral ranges. It means that various methods can be implemented simultaneously, which leads to the enhancement of the groups of harmonics in the shorter-wavelength spectral region and a single harmonic in the longer-wavelength spectral region. In other words, the number of generated coherent XUV photons of specific harmonic increases during the merger of some of these four approaches (RH effect, two-color pump, and nanoparticles). Meanwhile, the same harmonic or the same group of harmonics increases differently due to the step-by-step implementation of the four above-mentioned techniques in different spectral ranges.

The conditions for QPM were chosen in such a way that the harmonics around the 38th order (35 nm) were phase-matched, but not those around the 21st order (62 nm), where the resonance enhancement occurs. The equation *q*_QPM_ ∝ (*l* × *N*_e_)^−1^ allows expecting the tuning of the enhancement of a group of harmonics along the whole XUV spectrum. This equation assumes that, by using longer jets, one can tune the enhanced group of harmonics towards the region of enhancement of a single harmonic in the case of indium plasma (H21 for 1310 nm pumps and H13 for 800 nm pumps). Such a tuning, but only up to the 45 nm region was reported in a few publications (for example^78^). The maximally enhanced harmonics (*q*_QPM_) followed the rule when the product *l* × *N*_e_ was maintained constant at a fixed electron density of the silver plasma. Similar variations of harmonic spectra were observed in the case of manganese plasma. Unfortunately, the maximally enhanced harmonic could not be tuned towards the 62 nm spectral region where the enhanced 21st harmonic of the 1310 nm pump in the indium plasma was achieved.

Another technique to tune the range of enhanced harmonics by changing the conditions of target ablation was reported in Ref.^55^. The comparative studies of the harmonic spectra generated in the case of an 800 nm pump from the five-jet structure at different fluencies of 370-ps HP on the target surface were accomplished by changing the energy of heating radiation using the calibrated filters. In that case, a tuning of the maximum of the spectral envelope towards the higher-order harmonics with a decrease in the fluence of heating radiation was observed. Meanwhile, again as in the previous case, an increase in the fluence of HP did not lead to the tuning of the QPM conditions above the 35 nm spectral region.

To create the QPM conditions, one has to maintain a coupling between the driving and harmonic waves. For a given size of individual plasma jet (~ 0.5 mm), the QPM for the *q*th harmonic could be maintained at a fixed product *q* × *N*_e_. Correspondingly, a decrease of *N*_e_ at weaker excitation of the target should lead to the optimization of the QPM for higher *q*th to keep the product *q* × *N*_e_ unchanged at the fixed spatial characteristics of multi-jet plasma. Notice that this is an inconvenient method for tuning the group of enhanced harmonics. It also does not allow tuning the maximally enhanced harmonic towards the low-order harmonics (i.e. towards the range of 62 nm where the enhancement of a single 13th harmonic of 800 nm pump or 21st harmonic of 1310 nm pump can be achieved in the case of indium plasma).

The merger of the spectral ranges where the RH and quasi-phase-matching mechanisms coincide in the spectral scale was realized in the case of the enhanced single harmonic generated in the shorter wavelength region (45 nm) once the resonance enhancement and QPM were combined in the case of tin plasma using the near-infrared DP^79^. Unfortunately, the enhancement factor, in that case, was not as high as in the case reported in the present paper (3 and 50, respectively). This inefficient enhancement occurs due to a difference in the “optimal plasma” formation for two cases (RH-induced enhancement and QPM-induced enhancement). The former process requires lesser excitation of the target compared with the latter one to achieve a suitable realization of the enhancement mechanism.

As mentioned earlier, in the tunnel ionization regime, the mechanism of resonance harmonic generation for most materials can be explained by the four-step model^19^. The first two steps of this model are the same as the classical three-step model of HHG^3,4^, i.e., tunnel ionization of the valence electron and its acceleration within the continuum. The third step, however, involves resonant capture of the tunnel-ionized electron into the AIS, i.e., a discrete state embedded in the continuum, which is followed by the fourth step involving a radiative transition from the AIS to the initial ground state emitting resonance harmonic. This process is fixed for each element of the periodic table and cannot be tuned. Correspondingly, the only way to combine the spectral ranges of enhancement mechanisms is to tune the QPM process towards the shorter-wavelength region, which is impossible for the most of plasma species (particularly, indium plasma).

In the present case, the application of the four methods of harmonics enhancement was realized in a single set of experiments, though in different spectral ranges. The enhancement using resonance effect in plasma + enhancement using a two-color pump of plasma + enhancement attributed to the involvement of nanostructures was demonstrated in the case of the 21st harmonic (62 nm) of 1310 nm radiation, while the fourth method of enhancement through QPM was demonstrated for a large group of harmonics but in another, shorter-wavelength region of XUV (30–50 nm). Below the reasons why it was impossible to enhance the 21st harmonic using the QPM approach are discussed.

The relation *q*_QPM_ ∝ (*l* × *N*_e_)^−1^ correctly describes the link between the three parameters along the whole XUV spectral range. The problem is how to simultaneously fulfill the quasi-phase-matching-induced variation of the group of enhanced harmonic and the requirement to maintain a “suitable” (or “optimal”) ratio between the concentrations of neutrals (and ions) and free electrons in the plasma plume. Those two terms refer to the conditions when, from one side, the high concentration of neutrals (and singly-charged ions) prevails in the growth of harmonic yield over the growing influence of the free electrons strongly suppressing the phase-matching conditions, especially for the highest orders of generating harmonics. Therefore requirement #1 to not strongly decrease the phase matching conditions due to the presence of a large number of free electrons and requirement #2 to tune the maximally enhanced group of harmonics towards the lowest order of resonance harmonic (H21 of 1310 nm radiation in the case of indium plasma) cannot be realized simultaneously, at least at the presently used configuration of the experiment.

Intuitively one can assume that, following the *q*_QPM_ ∝ (*l* × *N*_e_)^−1^ relation, one should increase either the size of the single jet (or the width of the slit in the multi-slit mask) or the concentration of free electrons or both those parameters to increase the wavelength of the maximally enhanced harmonic. Here one has to take into account the impeding factors for HHG caused by the increase in the concentration of free electrons. Though the increase of heating pulse energy indeed leads to a decrease in *q*_QPM_^48^, this option has its limits. At larger fluencies of heating pulses, a larger number of free electrons appear. Moreover, simultaneously with those free electrons, a strong and broadband plasma emission produces at these conditions of laser ablation. The intensity of this emission rapidly exceeds the intensity of the harmonics. The plasma emission overlaps the whole harmonic spectrum and hardly allows further separation and application of this mixture of coherent and incoherent XUV radiation. Correspondingly, no information can be retrieved about the characteristics of the harmonic spectrum at these conditions.

As for the increase in the size of the jet, another restricting factor starts to play an important role. At the fixed length of extended and homogeneous plasma (5 mm in our case), the increase of the width of a single jet (or the width of the slit in MSM) will lead to a decrease in the number of plasma jets appearing on the target surface. For example, Fig. 3 shows that the maximally enhanced harmonic (H29) of 800 nm laser was obtained in the case of five 0.5 mm jets. These five jets fully cover the whole length of the extended 5-mm plasma (since one has to take into account a 0.5 mm distance between the jets). To shift *q*_QPM_ from H29 to H13 (the resonance harmonic of In plasma in the case of the 800 nm laser, which also corresponds to the H21 of the 1310 nm radiation) one has to increase the width of the slit from 0.5 to ~ 1.12 mm. It means that only less than three jets can be created using the 5 mm long plasma assuming the conditions when the width of slits becomes equal to the distance between them.

As has already been mentioned, the experiments with a variable number of slits were performed to demonstrate the quadratic dependence of the harmonic yield on the number of jets. An approximately two-fold increase in the width of slits will lead to the same decrease in the number of jets. Correspondingly, the overall harmonic yield becomes four times smaller. Apart from the notable decrease of the QPM-induced enhancement, these conditions hardly can be considered as those that pursue the advantages of the quasi-phase-matching effect. Moreover, to maintain the coupling of the driving and harmonics waves, the QPM has to start playing an important role when the number of jets allows an increase in the number of flips of the relative phases of interacting waves. It was shown in Ref.^55^ that the QPM is better realized using a large number of jets. Contrary to that, a lesser number of jets almost erase the QPM effect.

One can speculate that we can create a longer plasma (say, 20 mm long extended plume) by using the special focusing conditions, then use the MSM with broader slits and thus try to move *q*_QPM_ toward the lower-order harmonics. Notice that there are a few other obstacles, both technical and physical, which can prevent achieving this goal using the above geometry.

## Conclusions

The presented studies have shown that HHG in LIP using the joint implementation of different methods of harmonics enhancement can be used as an efficient source of radiation with photon energy in the range between 12 and 100 eV, corresponding to the 15–100 nm wavelength range. We demonstrated a method to generate intense, ultrashort pulses of XUV via implementation of the four mechanisms of HHG enhancement in different spectral ranges, which increase the resonant enhancement factor of the RH, allows generation of the group of enhanced higher-order harmonics due to the formation of the quasi-phase-matched conditions in the XUV range, involves the application of nanoparticles (instead of atoms and singly-charged ions) leading to the multi-fold growth of harmonic yield, and uses two-color-pump induced growth of harmonics yield. The 25- to 50-fold increase of harmonic yield, depending on the wavelength range, was demonstrated compared with the case when a single-color pump of atomic indium plasma was used.

## Data Availability

The datasets used and analyzed during the current study are available from the corresponding author upon reasonable request.
